# Design of a simplified cranial substitute with a modal behavior close to that of a human skull

**DOI:** 10.3389/fbioe.2024.1297730

**Published:** 2024-03-22

**Authors:** Natacha Elster, Johanna Boutillier, Nicolas Bourdet, Pascal Magnan, Pierre Naz, Rémy Willinger, Caroline Deck

**Affiliations:** ^1^ French-German Research Institute of Saint-Louis, Saint-Louis, France; ^2^ ICube Laboratory, Strasbourg University, Strasbourg, France

**Keywords:** experimental modal analysis, finite element model, human skull substitute, parametric study, vibrations

## Abstract

Individuals exposed to the propagation of shock waves generated by the detonation of explosive charges may suffer Traumatic Brain Injury. The mechanism of cranial deflection is one of many hypotheses that could explain the observed brain damage. To investigate this physical phenomenon in a reproducible manner, a new simplified cranial substitute was designed with a mechanical response close to that of a human skull when subjected to this type of loading. As a first step, a Finite Element Model was employed to dimension the new substitute. The objective was indeed to obtain a vibratory behavior close to that of a dry human skull over a wide range of frequencies up to 10 kHz. As a second step, the Finite Element Model was used together with Experimental Modal Analyses to identify the vibration modes of the substitute. A shaker excited the structure via a metal rod, while a laser vibrometer recorded the induced vibrations at defined measurement points. The results showed that despite differences in material properties and geometry, the newly developed substitute has 10/13 natural frequencies in common with those of dry human skulls. When filled with a simulant of cerebral matter, it could therefore be used in future studies as an approximation to assess the mechanical response of a simplified skull substitute to a blast threat.

## 1 Introduction

Individuals subjected to high-speed loadings such as detonating explosive charges can be injured in many ways depending on their distance from the explosion. The person may be blasted, riddled by munition fragments or surrounding materials, thrown against obstacles, and even burned if too close to the epicenter. The head can be severely affected by such events (Wojcik et al., 2010), particularly by the propagation of shock waves. To explain the observed cerebral lesions (Trudeau et al., 1998; Armonda et al., 2006; Hoge et al., 2008; Warden et al., 2009; Hicks et al., 2010; Rosenfeld et al., 2013; Magnuson and Ling, 2018), several injury mechanisms were proposed in literature. One of those is of particular interest: the cranial deflection. This hypothesis implies that the load applied when shock waves reach the human skull can induce brain lesions due to compression and shear loading on the underlying cerebral matter (Bolander, 2011; Chandra and Sundaramurthy, 2015).

Literature describes several geometric human head substitutes developed to investigate this specific phenomenon. Dal Cengio Leonardi et al. (2011) filled Synbone spheres with either Sylgard gel (527 A&B) or aqueous glycerin; Goeller et al. (2012) created an ellipsoidal model in polycarbonate and filled it with either water or Sylgard gel 527; and finally Hua et al. (2014) designed a polycarbonate spherical shell filled with water. Those substitutes were then instrumented with pressure sensors inside their shell, while strain gauges were glued to their outer surfaces. Shock waves of varying durations between 0.4 ms and 6.0 ms, with maximum amplitudes ranging from 40 kPa to 210 kPa were applied to each substitute. Several metrics of interest were collected such as the time of wave arrival, the first peak values of the internal pressures and the shell strains, as well as the extreme values. Results showed that internal pressures and shell strains are related; however, only time-domain analyses were performed in those mentioned studies (Elster et al., 2023). Since the time history of quasi-ideal blast waves could be approximated by pulse signals with broad frequency spectra, it would be interesting to analyze blast related results in the frequency domain as well. Indeed, the shock front could excite a large range of cranial vibration modes. It would therefore be worth using Experimental Modal Analyses (EMA) to characterize the vibration modes of the head substitutes beforehand, and then as a second step to compare the obtained results with the frequencies extracted from the strain signals recorded during blast events.

In Fujiwara et al. (1989) a cranial substitute able to reproduce the first vibration mode shape of a dry human skull was designed. The current study aims to create a new skull substitute that could replace a human one for frequency-domain analysis, considering a larger number of natural frequencies than in Fujiwara’s study. Indeed, EMA has been performed on four 50th percentile dry human male skulls in the literature (Franke, 1956; Gurdjian et al., 1970; Khalil et al., 1979; Taleb et al., 1993). In the range [0–5] kHz, 13 natural frequencies were found in all experiments combined. The present study aims to design a new cranial substitute based on these frequencies.

To reach this goal, the manufacturing method of the substitute must first be selected and the mechanical properties of the material need to be characterized. Secondly, the geometry and dimensions of the substitute are then adapted to approximate a 50th percentile dry human skull, while maintaining a close vibratory response. The third part is dedicated to simultaneously perform Numerical and Experimental Modal Analyses to identify all vibration modes of the substitute. Finally, the obtained results are compared in terms of frequencies with those described in the literature on dry human skulls to evaluate the relevance of the new cranial substitute.

## 2 Material and methods

**FIGURE 1:**
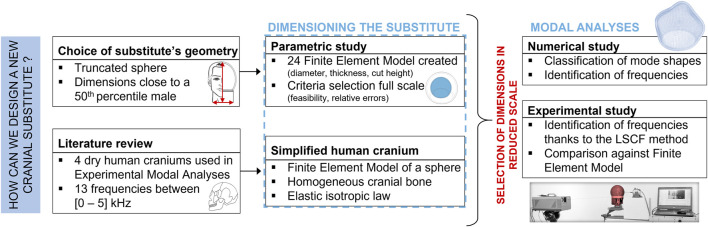
Diagram of the methodology followed to design a human skull substitute.

The methodology adopted to design the new cranial substitute is summarized in Figure 1. A simplified geometry was chosen to approximate the human skull: a hollow truncated sphere. For enhanced feasibility and cost-effectiveness, an additive manufacturing process was thus selected: Polyjet 3D-printing technology (@Stratasys, 2019). The material chosen is the IORA Black resin developed by the firm iSQUARED^2^, whose mechanical properties are far from those of the cranial bone in terms of Young’s modulus, density and Poisson ratio (Elster et al., 2022). Hence, since the substitute is meant to have a vibratory response akin to that of a dry human skull, a parametric study must be performed on its dimensions to compensate for the existing disparities. In addition, a reduced scale was considered to select the optimal geometry due to experimental constraints of the blast testing field. Finally, once the dimensions have been settled, numerical and experimental studies are carried out simultaneously to characterize the vibratory response of the new substitute, and to compare its behavior with literature data.

### 2.1 Parametric study

**FIGURE 2:**
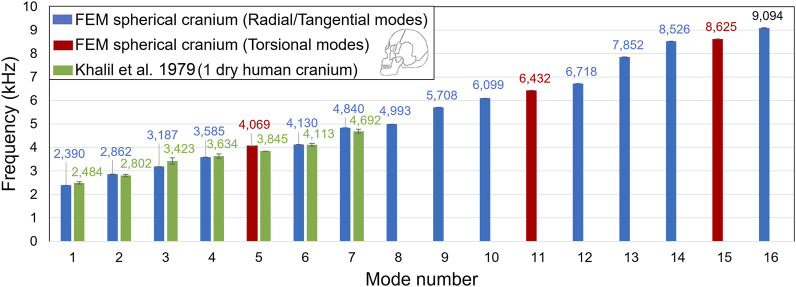
Comparison of the numerical frequencies computed from a FEM of a spheric-approximated human skull to those measured experimentally on a dry human skull of the 50th percentile in the literature (Khalil et al., 1979).

The first step consists in creating a Finite Element Model (FEM) of a sphere approximating a human skull. This sphere has an outer diameter of 180 mm like the skullcap (Got et al., 1983; Bushby et al., 1992; Haeussinger et al., 2011), and a constant thickness of 7 mm to mimic the frontal cranial bone (Got et al., 1983; Lynnerup, 2001; Haeussinger et al., 2011). For the purpose of simplification, the cranial bone is assumed to be made of a homogeneous and isotropic material, following a linear elastic behavior law with a Young’s modulus of 5,000 MPa (Verschueren et al., 2006; Delille, 2007; Auperrin, 2009), a Poisson ratio of 0.2 (McElhaney et al., 1970; Nishimoto et al., 1995) and a mass density of 1,700 kg/m^3^ (McElhaney et al., 1970; Nishimoto et al., 1995). The LS-Dyna software is used to mesh the structure, using 30996 hexahedral solid elements with 3 element layers through thickness, and an average element size of 3.1 mm. The implicit solver with the element formulation #18 is selected. This formulation is tailored for hexahedral elements with eight integration points during an implicit linear resolution (LS-DYNA R11 Keyword Manual Volume I, 2018). Free boundary conditions are imposed. In the frequency range [0–10] kHz, 16 vibration modes are computed for the spherical model: 3 torsional modes and 13 radial/tangential modes. This FEM is validated according to the vibration theory of thin spherical shells (Wilkinson, 1966): the relative errors calculated between the FEM and analytical natural frequencies are ranging from 0.01% to 0.93%. Moreover, Figure 2 compares the frequencies of the FEM with experimental results described by Khalil et al. (1979), where a human skull was struck by an impact hammer, resulting in an impulse signal between 20 Hz and 5,000 Hz. The first seven frequencies of the FEM are close to several frequencies measured on the dry human skull, with relative errors ranging from 0.4% to 6.9%.

In future studies, the newly developed substitute is intended to be exposed to the detonation of explosive charges in free-field conditions. The experimental ground where the detonations will be carried out has limitations. These include the maximum admissible explosive mass and the allowable distances between the instrumented substitute and the center of the detonation. Given the exposure parameters being sought, it is essential to work in a reduced scale of 5/6 ratio. The similarity laws and scale effects will have to be applied in future blast tests. In the context of explosives, the similarity laws indicate that reduced-scale studies can be employed to ascertain the characteristics of a full-scale blast wave, and *vice versa* (Baker et al., 1983). Additionally, scale effects extend to the displacement of the exposed structure (Baker et al., 1991).

With this in mind, the dimensions of the new cranial substitute have to be selected. Working with a hollow truncated sphere, three parameters have to be defined: the external diameter of the shell, its thickness, and its height. A parametric study is thus performed to evaluate the influence of each parameter on the natural frequencies of the substitute. Several design criteria must be taken into account to define the possibilities of each setting. First, the substitute has to be large enough to contain the sensors, while being smaller than 148 mm in diameter, which is the size of the printing plate. Four external diameters are chosen: 120 mm, 125 mm, 133 mm and 142 mm. In addition, based on a preliminary study, a minimum thickness of 5 mm in reduced scale is required for the substitute to withstand a blast. Therefore, the thicknesses chosen are 5 mm, 5.8 mm and 7.5 mm. Finally, three truncation ratios are defined: 5/8, 3/4, and 7/8 of the volume. As a result, twenty-four combinations of dimensions were tested during the parametric study. To select the most suitable combination, three selection criteria were applied: the physical feasibility, and the relative and absolute gaps in terms of frequencies against the spheric-approximated skull FEM values. Ultimately, the chosen dimensions are: an external diameter of 133 mm, a thickness of 5 mm, and a height of 108 mm (Elster et al., 2022).

### 2.2 Finite Element Model of the substitute

**FIGURE 3:**
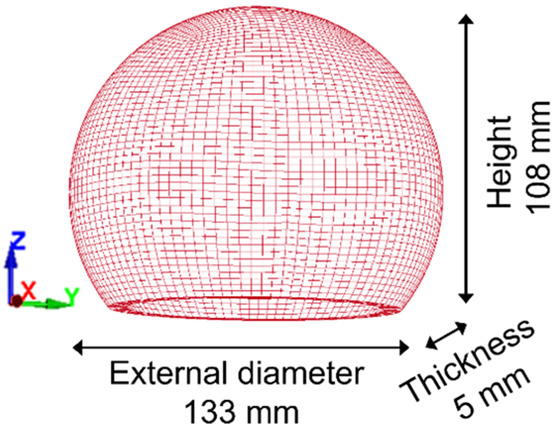
Illustration of the Finite Element Model of the skull substitute.

After selecting the optimal dimensions, the FEM of the new skull substitute is created, as illustrated in Figure 3. The model is meshed with 14400 hexahedral elements of average size 3.1 mm with 3 elements through thickness. Again, element formulation #18 is used, with an elastic isotropic constitutive law. Nodes located at the bottom of the FEM are embedded. The material properties of the IORA Black resin were determined experimentally in a previous study, giving a Young’s modulus of 2,663 ± 228 MPa, a Poisson ratio of 0.38 ± 0.04, and a mass density of 1,170 kg/m^3^ (Elster et al., 2022).

Computed vibrations modes can be visually classified into four different types: torsional, biaxial, radial, and axial modes. Radial and axial modes can be further described using the thin-shell theory (Rossing, 1982; Xie et al., 2017; Du et al., 2019), for which two parameters (n, m) are defined: n represents the number of symmetry lines passing through the center of the cavity; and m is the number of circles that can be drawn in top view.

### 2.3 Experimental Modal Analysis of the substitute

An Experimental Modal Analysis aims to extract the vibration modes of a structure. The structure under test is subjected to external forces through a frequency sweep in given input points; while resulting vibrations are recorded at strategic locations called outputs (Ewins, 2000). A modal identification method then exploits the experimental signals and determines the associated natural frequencies and mode shapes of the structure.

**FIGURE 4:**
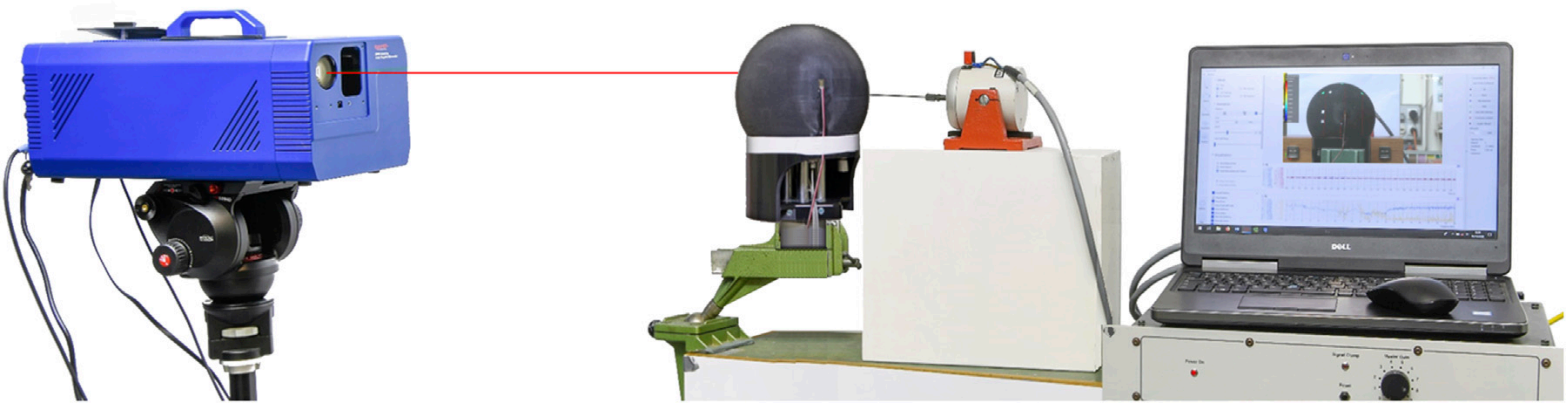
Setup used to perform an Experimental Modal Analysis on the 3D-printed prototype of the human skull substitute.

In this study, a prototype of the skull substitute is 3D-printed using the Polyjet technology, applying resin droplets to the printing plate layer by layer. Each layer is then immediately cured by UV radiation and the structure can be used immediately after printing. Theoretically, the molecular bonds between the layers are maintained and material properties are preserved both in the printing plane and in thickness, allowing assumptions of isotropy and material homogeneity to be considered (Williams et al., 2011). In the current study, the in-fill density was 100% for a layer thickness of 0.028 mm. The skull substitute is then glued on a Dural plate (AU4G), circular in cross-section and 5 mm thick, using notches for attachment. A transition piece printed in the same material as the substitute is bonded directly to the substitute thanks to epoxy glue. The dimensions of this part are close to the neck in reduced scale: it is an open cylinder with a diameter of 120 mm and a length of 90 mm (Gordon et al., 1988; Vasavada et al., 2008). The prototype is finally clamped at its base and subjected to an EMA using the setup illustrated in Figure 4. An up-chirp signal of amplitude 2V and frequency ranging from 5 Hz to 12 kHz is transmitted to a shaker (LDS V200), whose metal rod is in contact with the substitute near its midline. A scanning laser vibrometer (OptoMET SWIR) is used to record the time history of the vibration velocity in a given output point at a recording speed of 49 mm/s and a sampling rate of 51.2 kS/s. Ten repetitions are conducted for each measurement to ensure the reproducibility of results.

**FIGURE 5:**
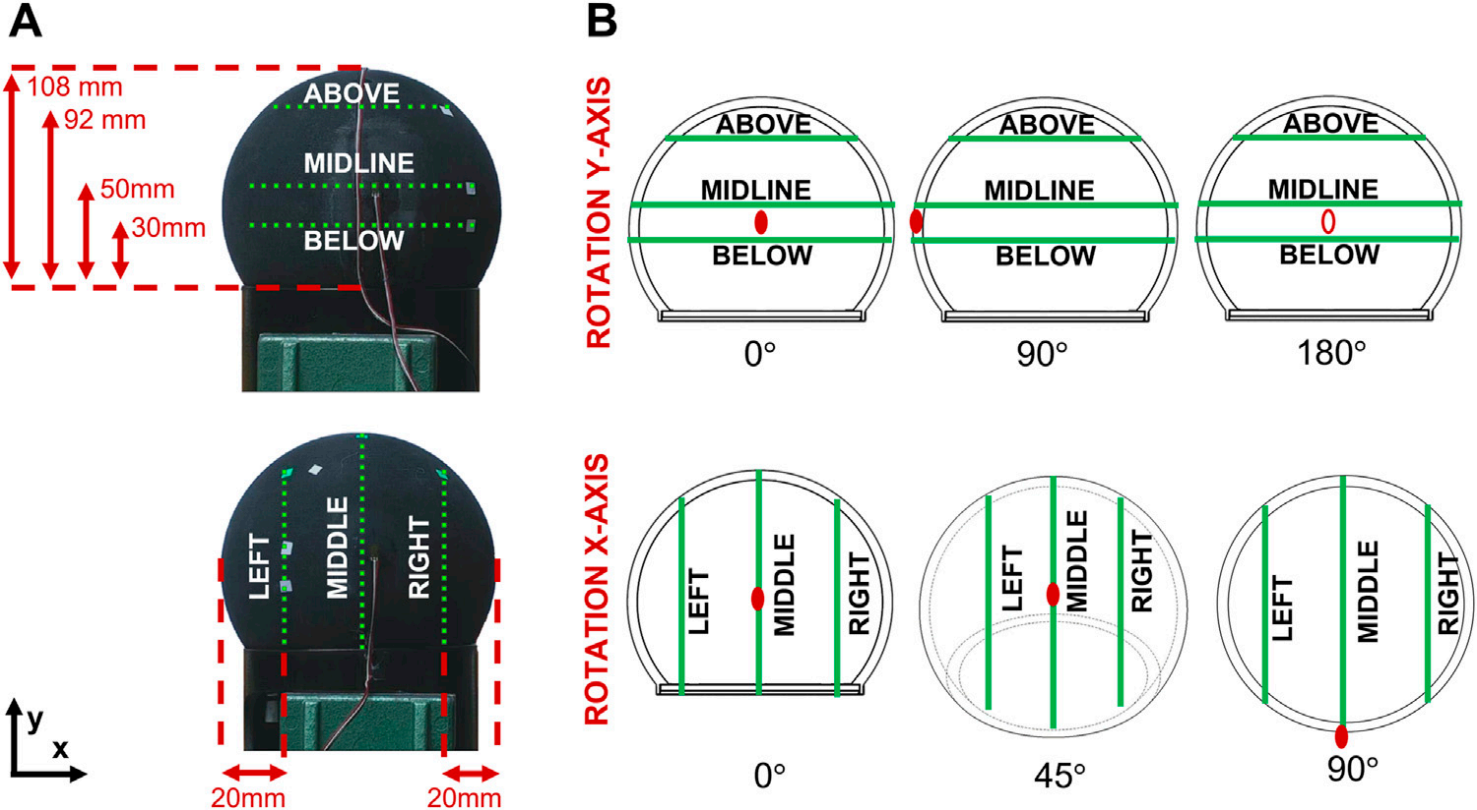
Positioning of the 3D-printed prototype in the Experimental Modal Analyses. **(A)** Horizontal and vertical measurement lines. **(B)** Six possible rotations along the x- and y- axes. The red dots show the rotation of the substitute.

To limit the number of outputs, six measurement lines are defined, as illustrated in Figure 5A: three horizontal lines “top”, “midline”, “bottom”; and three vertical lines “left”, “middle”, “right”. Taking symmetries into account, the substitute is then turned six times to reconstruct data for the entire structure in three dimensions (Figure 5B). For each position, the input point is at the extreme left on the midline. On average, 51 measurement points are defined per rotation.

**FIGURE 6:**
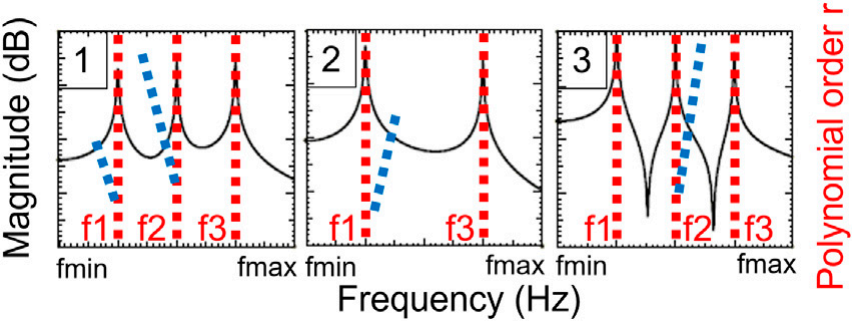
Stabilization diagrams for three theoretical measurement points using the Least Squares Complex Frequency method. The dotted lines in red represent the stable roots, and in blue the unstable roots. The graph shows the history of the data magnitude versus frequency.

The experimental data measured during the vibration tests are post-processed using a moving average filter with a kernel of 3 points. A modal identification algorithm is then applied to extract the parameters of the vibration modes: the Least-Squares Complex Frequency (LSCF) algorithm in output-only conditions is used (Verboven, 2002; Peeters and Van der Auweraer, 2005), considering a Single Input Single Output system for each measurement point. First, the velocities recorded at the output points are computed in the frequency-domain to create stabilization diagrams, as illustrated for three theoretical measurement points in Figure 6 (black curves). To robustly identify each frequency, p polynomials are generated to describe the frequency evolution at each measurement point. For every polynomial r, where 1 < r < p, its roots are plotted directly on the frequency evolution against the polynomial order, here in red (stable roots) and blue (unstable roots). Stable roots form a stabilization line, indicating a natural frequency of the structure; while unstable roots are purely mathematical. Figure 6 plots the magnitude against frequency for three points, showing that amplitude peaks correspond to stabilization lines.

From the stabilization diagrams, the natural frequencies can be extracted using two criteria: the amplitude spectrum with a tolerance of ±25 Hz, and the stabilization lines showing the convergence of the roots towards the frequencies with increasing polynomial order, where the maximal polynomial order p is 300. When all conditions are satisfied, the frequency is selected. Associated mode shapes are then constructed from the imaginary part of the measured data.

## 3 Results

This section presents the results of modal analyses carried out first numerically thanks to a FEM of the new cranial substitute; and second experimentally on the 3D-printed prototype. Both analyses are conducted simultaneously to characterize the vibration modes of the substitute, while eliminating spurious experimental data and validating the FEM at the same time. Mode shapes are first identified by comparing the measured experimental deformations with the FEM computed cut lines amplitudes. Once the numerical and experimental pairing is established, the natural frequency identification errors are quantified to assess how close both models are.

### 3.1 Identification of mode shapes

#### 3.1.1 Numerical results

First of all, looking at the FEM mode shapes gathered in Table 1, 58 numerical vibration modes are identified in the frequency range [0–10] kHz: 3 torsional modes, 5 biaxial modes and finally 50 radial modes, 8 of which are axial. The torsional modes are scattered over the spectrum, the first one being at 2,272 Hz (mode 3), then 6,303 Hz (mode 29), and 9,686 Hz (mode 56). The same applies to the biaxial modes, since the first is found at 4,278 Hz (mode 13) and the last at 9,069 Hz (mode 50). As the thickness of the substitute’s shell is small compared to its diameter (5 mm thick for an external diameter of 133 mm), the so-called radial modes, for which the deformations observed are predominantly normal to the surface, can be described by the parameters (n, m) according to the thin-shell vibration theory (Rossing, 1982; Xie et al., 2017; Du et al., 2019). For axial modes, n equals 0 and m varies between 1 and 8; while the predominantly radial modes have a parameter n between 1 and 9; for a parameter m varying between 0 and 8.

**TABLE 1:** Computational mode shapes of the Finite Element Model of the skull substitute in the range [0–10] kHz displayed in isometric view.

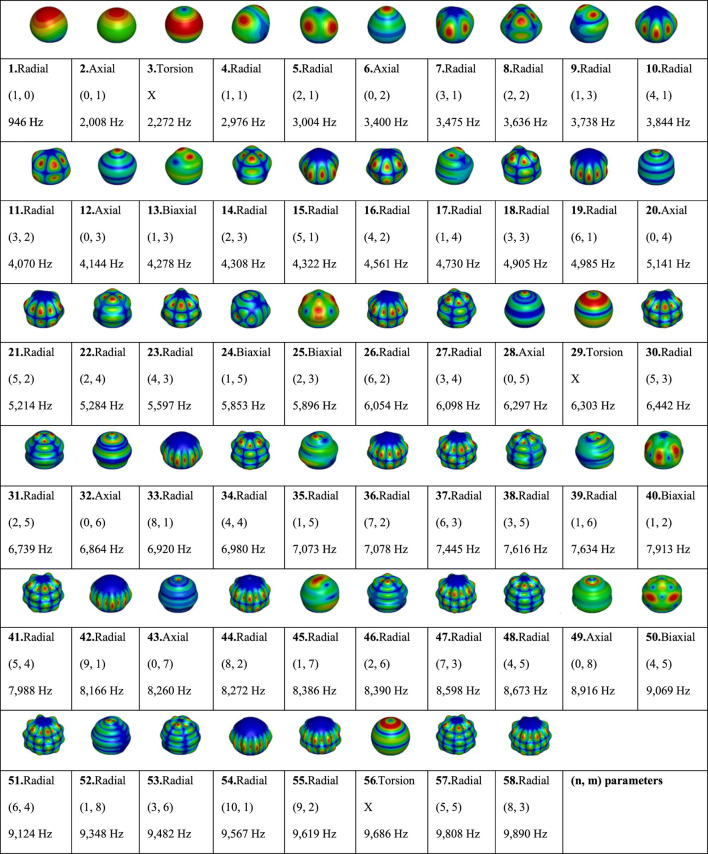

Parameters (n, m) respectively describe the number of lines of symmetry passing through the center of the cavity; and the number of circles in top view.

#### 3.1.2 Experimental results

**FIGURE 7:**
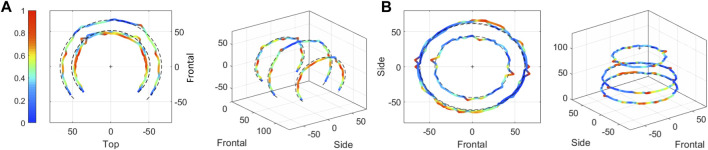
Example of a “complete” mode shape obtained during the Experimental Modal Analysis of the 3D-printed prototype. The calculated natural frequency is 2,953 ± 26 Hz. Color coding is the normalized magnitude of vibrations for each mode shape, and the measurement lines are defined in Figure 5. **(A)** Rotations of the vertical measurement lines along the x-axis in side and isometric views. **(B)** Rotations of the horizontal measurement lines along the y-axis in top and isometric views.

The mode shapes corresponding to the 51 experimentally detected frequencies are depicted. Only 23 of them, i.e., 45%, are “complete” mode shapes, meaning that the same frequency is detected for each of the three rotations around either the x or y axis. Figure 7 shows an example of a complete mode shape detected at 2,953 ± 26 Hz, with deformations recorded for the x-axis rotations of the vertical measurement lines in side and isometric views (A); and those obtained during the y-axis rotations of the horizontal measurement lines in top and isometric views (B). The colors represent the magnitude of the measured deformations in normalized values, where the maximum magnitude is shown in red and the minimum in blue.

**FIGURE 8:**
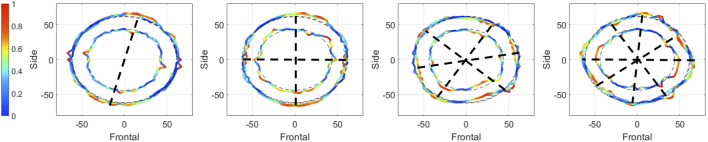
Example of four mode shapes obtained during the Experimental Modal Analysis of the 3D-printed prototype. The mode shapes are detected during rotations of the horizontal measurement lines along the y-axis and are represented here in top view. The black dotted lines represent the symmetry lines defining the parameter n. Color coding is the normalized magnitude of vibrations for each mode shape, and the measurement lines are defined in Figure 5.

In a second step, the 2D complete and incomplete experimental mode shapes are analyzed to identify the parameters (n, m) of the radial modes. As an example, Figure 8 shows four modes shapes detected during rotation of the horizontal measurement lines along the y-axis in top view. Color coding once again represents the magnitude of vibrations in normalized values. The symmetry lines drawn in black are deduced from the amplitude variations of the cut lines. The illustrated mode shapes have a parameter m equal to 1 for a parameter n varying between 1 and 4. Ultimately, 34/51 experimental mode shapes are formally identified using (n, m) parameters, representing 67% of the detected experimental frequencies. It should be noted that out of these 34 natural frequencies, 14 of them are associated in pairs with the same vibration mode, which implies that 7 modes are detected twice (once during x-rotations and once during y-rotations). Thus 27 vibration modes are formally authenticated experimentally for the 3D-printed prototype: 3 biaxial modes, 0 torsional modes and 24 radial modes.

#### 3.1.3 Numerical and experimental comparison

**FIGURE 9:**
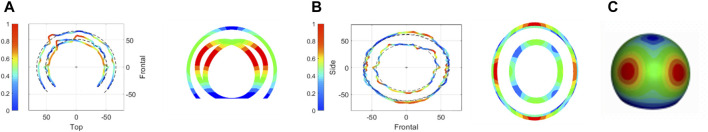
Comparison of experimental and numerical mode shapes of the substitute’s vibration mode (n = 2, m = 1) at the natural frequency 3,139 ± 30 Hz. Color coding is the normalized magnitude of vibrations for each mode shape. **(A)** Side view. **(B)** Top view. **(C)** Isometric view.

Finally, a comparison between experimental and computational data is required. Each experimental mode shapes are compared with computational ones in order to both validate the FEM and eliminate spurious data from the experimental set. An example is presented in Figure 9 for the mode (n = 2, m = 1). The experimental frequency is 3,139 ± 30 Hz, against 3,004 Hz for the FEM. In side view, a shift is observed between the left and right parts (A); while in top view, the largest cut lines display four parts with high amplitudes of vibrations (B). All comparisons between experimental and numerical sets are presented in the Supplementary Data SA as 2D plots along either the x or y axis of rotation. The color coding represents the mode shapes amplitudes in normalized values per set.

### 3.2 Quantification of errors in terms of natural frequencies

After matching the experimental and numerical data through visual identification of the mode shapes, errors made on the natural frequencies are quantified. Table 2 compares the results of the FEM and 3D-printed prototype of the skull substitute. In total, 58 vibration modes were computed with the FEM and 51 were found experimentally. By comparing the two data sets, 34 common modes (including 7 detected twice) were formally authenticated: 3/5 bi-axial modes, 0/3 torsional modes and 24/50 radial modes. The computed relative errors are small (0.3%–5.7%), corresponding to absolute errors between 18 Hz and 280 Hz. Unfortunately, it is clear that not all vibration modes were detected using the experimental protocol outlined in this study. The factors that may explain this shortcoming will be discussed further below.

**TABLE 2:** Comparison of the natural frequencies of the Finite Element Model and the 3D-printed prototype of the cranial substitute.

Parameters (n, m)	Experimental frequency (HZ)	Computational frequency (HZ)	Relative gap (%)	Absolute gap (HZ)
(0, 1)	2,102 ± 23	2,008	4.7	94
(1, 1)	2,953 ± 26	2,976	0.8	23
(2, 1)	3,139 ± 30	3,004	4.5	135
(3, 1)	3,512 ± 52	3,475	1.1	37
(2, 2)	3,610 ± 36	3,636	0.7	26
(1, 3)	3,824 ± 83	3,738	2.3	86
(4, 1)	3,954 ± 66	3,844	2.9	110
Bi-axial	4,145 ± 97	4,278	3.1	133
(5, 1)	4,344 ± 79	4,322	0.5	22
(2, 3)	4,371 ± 21	4,308	1.5	63
(4, 2)	4,587 ± 78	4,561	0.6	26
(6, 1)	5,168 ± 60	4,985	3.7	183
(3, 3)	5,185 ± 98	4,905	5.7	280
(2, 4)	5,302 ± 27	5,284	0.3	18
Bi-axial	5,698 ± 116	5,853	2.7	155
(6, 2)	6,081 ± 95	6,054	0.4	27
(3, 4)	6,126 ± 100	6,098	0.4	28
(5, 3)	6,642 ± 156	6,442	3.1	199
(4, 4)	6,939 ± 13	6,980	0.6	41
(1,5)	7,175 ± 113	7,073	1.4	102
(6, 3)	7,221 ± 60	7,445	3.0	224
(5, 4)	7,914 ± 93	7,988	0.9	75
(7, 3)	8,448 ± 139	8,598	1.8	150
(4, 5)	8,604 ± 107	8,673	0.8	69
Bi-axial	8,885 ± 4	9,069	2.0	184
(6, 4)	8,921 ± 162	9,124	2.2	203
(1, 8)	9,223 ± 28	9,348	1.3	125

## 4 Discussion

At the end of this study, some issues still need to be addressed. First, on which frequency interval has the vibratory behavior of the new cranial substitute been evaluated? Second, could this substitute be used instead of dry human skulls for high-speed loading experiments? Finally, the outlook of this study will be discussed, including the use of a brain simulant in conjunction with the skull substitute for blast loadings.

### 4.1 What is the validity of the modal analyses performed on the substitute?

The quantification of disparities between the experimental and numerical vibration modes of the cranial substitute evidenced a good fit between the FEM and the 3D-printed prototype. However, it is worth noting that not all vibration modes were detected experimentally, mainly because of the experimental setup. A force was applied to the structure using a shaker and a metal rod instead of an impact hammer to ensure a broad frequency range that is easily reproducible. The electrical signal transmitted from the signal generator to the shaker was an up-chirp with a constant amplitude of 2V and a frequency varying between 5 Hz and 12 kHz. Since the applied input signal remains unknown, merely output-only conditions could be set for the modal identification step. Hence, only the magnitude of the recorded velocities could be exploited, and not the magnitude, phase, and coherence function information, as opposed to using the LSCF algorithm with input-output conditions. To obtain more information during vibration tests, the input signal could be measured by positioning a triaxial accelerometer in the vicinity of the input point. Furthermore, the use of a laser vibrometer simplifies the modal analysis, as it is only necessary to define a set of measurement points to start an automatic analysis. While most radial modes are effectively detected using this method, biaxial modes pose a greater challenge, and torsional modes cannot be formally identified because the laser points perpendicular to the outer surface of the substitute. Once again, the use of a triaxial accelerometer could have been useful for these modes, but as there were fewer of them, the choice fell on the ease of implementation, the non-addition of the accelerometers’ masses, and the simplicity of the results’ processing.

Another factor to consider when discussing the validity of the modal analyses performed is the sensitivity of the numerical results to the mechanical properties of the material. A sensitivity analysis revealed that a variation of ±10% in Young’s modulus causes a uniform frequency shift of ±4% in the direction as the modulus change. Additionally, a change of ±20% in the Poisson ratio leads to more significant non-uniform variations, reaching up to 15%. Notably, this variation affects the time of emergence of certain vibration modes, especially as the material approaches incompressibility. Plus, structural damping cannot be implemented in the current study, which explains a plausible frequency shift of the calculated vibration modes.

The disparities quantified during the comparison of the two modal analysis datasets (experimental and computational) can be further explained by several factors. First, despite the moving average filtering applied to the signals; high-frequency noise taints the dataset. These uncertainties can lead to identification errors when using the LSCF algorithm to select the natural frequencies that are compared to the FEM. Another source of bias can arise from the stage of matching experimental and numerical data, as this relies mainly on the visual appearance of the mode shapes.

To assess whether the FEM is a good fit to the experimental data despite the observed discrepancies, the literature is consulted. Schedlinski et al. (2004) performed an EMA on a car body in white and then created a FEM. The model validation was performed between 0 and 100 Hz: of the 42 modes computed with the numerical model, 17 experimental modes were found to be common. The numerical model was then recalibrated using Young’s modulus. A similar approach was adopted by Sellami et al. (2017) for the validation of their FE model of a wind turbine in free vibration over the range [0–250] Hz. Numerically, 15 vibration modes were computed over this range, while 7/15 modes were detected with an impact hammer and 15/15 modes with the use of an accelerometer and a shaker. The study by Frankovský et al. (2022) created the FE model of a flat rectangular steel plate. The model was validated between 0 and 2000 Hz using only the first six experimental vibration modes. Hence, taking into account previously outlined experimental limitations, the Finite Element Model (FEM) of the skull substitute can be considered to be validated. This is further supported by the large number of vibration modes considered and the broad frequency range of interest. Indeed, more than 65% of the experimental data agree with the numerical results between 0 and 10 kHz. Plus, low relative and absolute errors were computed between the two Modal Analysis techniques, although only six measurement cut lines were considered.

### 4.2 Does the new substitute have a vibratory response close to dry human skulls?

The main objective of this newly designed cranial substitute is to reproduce the vibratory response of the human skull under high-speed loading. Unfortunately, in the literature, only four published studies describe EMA performed on 50th percentile dry human skulls (Franke, 1956; Gurdjian et al., 1970; Khalil et al., 1979; Taleb et al., 1993). In these experiments, a total of four male skulls were excited by an impact hammer whose signal varied in frequency from 0 Hz to a maximum of 5 kHz. The induced vibrations were measured by an accelerometer placed at various locations. In the end, a total of 13 natural frequencies were measured, all bibliographic studies combined. Khalil et al. (1979) detected the majority of these natural frequencies (11/13) on a single male skull. Only this particular study recorded frequencies above 1.5 kHz. There is also a significant discrepancy between the various bibliographic data. This can be partly explained by the boundary conditions used (simple-support, free boundary conditions, unspecified), the instrumentation of the skull, but also by the high variability of the mechanical properties of the skull, which depends on the age of the subjects and the preservation of the specimens (Fallenstein et al., 1969). Due to incomplete data on the rare mode shapes available (Khalil et al., 1979; Taleb et al., 1993), they cannot currently be compared with the data presented in the study. Therefore, the results can here only be matched in terms of frequencies because of the lack of modal analyses performed on human skulls in the literature. This limits the validation of the spheric-approximated skull FEM presented in Section 2.1; as well as the comparison with the 3D-printed prototype of the human skull substitute.

Table 3 compares several numerical and experimental natural frequencies of the substitute to those described in the literature. Of the 13 frequencies associated with dry human skulls, 10 are common to the FEM, and 8 are common to the 3D-printed prototype. These 8 experimental frequencies between 2 kHz and 5 kHz are close to the ones detected by Khalil et al. (1979) on a dry skull. This stems from the fact that, during the design of the skull substitute with the parametric study, the chosen dimensions were adjusted to favor Khalil’s frequencies. Indeed, a wide-range excitation signal was used in his study, thanks to an impulse generated by an impact hammer within a frequency range of [20–5,000] Hz. A large number of measurement points was also considered, representing more than 100 outputs, compared to 1 output for Franke (1956), 3 outputs for Gurdjian et al. (1970) and 29 outputs for Taleb et al. (1993). Regarding the comparison between the current skull substitute and the bibliographic data, relative errors were calculated for the natural frequencies, ranging from 0.7% to 9.9%. They can be attributed to several factors that can affect the overall vibratory response. First, the skull substitute weighs 245 g against almost 390 g for the cranial vault and around 800 g for an entire skull (Got et al., 1983). The vibration modes of a structure depend on its mass, rigidity and damping matrices. A variation in those properties lead to a modification of the observed mode shapes, and more specifically to a shift in frequency when mass is added or removed. Therefore, a parametric study was required to select the geometry of the skull substitute. This was essential to compensate for the observed disparities, attributed to the known differences in mechanical properties between the cranial bone and the IORA black resin. The most appropriate geometry for the substitute was a hollow truncated sphere in this case. However, the geometry evidently influences the obtained results even though it was dimensioned such as a maximal number of its natural frequencies were common with dry human skulls. Indeed, the substitute has a spherical shell, while the skull is made up of several bones, notably the cranial vault, the mandible and the maxilla, that are characterized by their sutures, crevices, and cavities. Furthermore, the vibratory response of a structure depends on its macroscopic composition. In the current study, the 3D-printed prototype is made of an homogeneous material, yet the cranial bone is composed of several layers (Saladin, 2003) that have specific material properties: the diploë as well as the inner and outer tables of the cortical bone. Lastly, the prototype of the cranial substitute was embedded at its base; but in the study carried out by Khalil et al. (1979), the dry skull was placed in simple support conditions. Varying the boundary conditions causes a change in the number of degrees of freedom at the contact section, and thus modify the vibration modes.

**TABLE 3:** Comparison of frequencies (Hz) identified on the substitute using Finite Element Modelling and Experimental Modal Analysis with those found on dry human skulls in the literature.

FEM	EMA	Dry skulls	Number of skulls (references)]		Experimental errors (%)
X	X	700	1	Taleb et al. (1993)	X
946	X	866 ± 42	3	Franke (1956); Gurdjian et al. (1970); Taleb et al. (1993)	X
X	X	1,342 ± 59	3	Khalil et al. (1979); Taleb et al. (1993)	X
X	X	1,770 ± 20	1	Khalil et al. (1979)	X
2,008	2,102 ± 23	1,893 ± 14	1	Khalil et al. (1979)	9.9
2,272	X	2,484 ± 47	1	Khalil et al. (1979)	X
2,976	2,953 ± 26	2,802 ± 58	1	Khalil et al. (1979)	5.8
3,475	3,512 ± 52	3,423 ± 130	1	Khalil et al. (1979)	2.5
3,636	3,610 ± 36	3,634 ± 96	1	Khalil et al. (1979)	0.7
3,844	3,954 ± 66	3,845	1	Khalil et al. (1979)	2.8
4,278	4,145 ± 97	4,113 ± 62	1	Khalil et al. (1979)	3.9
4,308	4,371 ± 21	4,284 ± 63	1	Khalil et al. (1979)	2.0
4,561	4,587 ± 78	4,692 ± 87	1	Khalil et al. (1979)	2.9

This research is not the first one attempting to reproduce the vibratory response of a dry human skull. The study by Fujiwara et al. (1989) is particularly noteworthy since they created a human skull substitute filled with degassed water that had a first vibration mode resonating at 500 Hz. They could reproduce part of the first mode shape of a dry human skull they had placed in simple support conditions. In this study however, only the first vibration mode was studied and the skull substitute was filled with a cerebral matter simulant, thus increasing the mass of the structure and thereby modifying its vibratory response. In the current study, despite the use of a very simple and approximate geometry for a new skull substitute, a good number of its natural frequencies are in common with those of dry human skulls, thus fulfilling the requirements.

### 4.3 Towards the design of a head substitute

As stated in the introduction, the ultimate goal of this research is to investigate the cranial deflection hypothesis. This hypothesis is one of the plausible injury mechanisms that could explain the occurrence of brain damage in individuals exposed to explosions. It could be investigated by studying the outer surface deformation signals of the subject in both time and frequency domains. To this end, a new human skull substitute with a simplified geometry was designed to ensure the reproducibility of results.

Although the created substitute meets those specific requirements, it is important to highlight that the design considered only the vibratory behavior of a human skull. Additional steps are required to incorporate skin and brain simulants, among others, to develop a head substitute that could be used to further investigate other injury mechanisms. The most commonly used brain simulant for these kind of studies is the ballistic gel (Ganpule et al., 2016; Salzar et al., 2017). This material replicates the properties of soft tissues during impacts (Jussila, 2004), but its preservation conditions depend on both the duration and the temperature (Cronin and Falzon, 2011). The sylgard gel is another simulant frequently used (Merkle et al., 2009; Dal Cengio Leonardi et al., 2011; Goeller et al., 2012), even though there is once again a limitation on the preservation duration after hardening. To overcome these issues while maintaining the proper density of brain tissue, some authors prefer turning to saline solutions (Salzar et al., 2017) or water (Goeller et al., 2012; Hua et al., 2014; Josey et al., 2016; Banton et al., 2018). One study stands out: an anthropomorphic head substitute was created by replicating the skin with urethane rubber, skull with polyurethane, cerebrospinal fluid with water and finally the brain with a silicone gel (Ouellet et al., 2012). However, shell deformations were not recorded in this study, and the vibratory response of the substitute was not assessed.

Adding the soft tissues will consequently lead to a modification of the vibratory behavior of the substitute. Hence, supplementary computational and experimental modal analyses will be required. Unfortunately, the literature lacks experiments carried out *in-vivo* for future comparison. One study involved volunteers who were impacted in the frontal zone, while accelerometers were placed on their occipital, vertex and temporal bones (Gurdjian et al., 1970). Three natural frequencies were measured *in-vivo*: 300 Hz, 560 Hz and 920 Hz. In addition, six *in-vivo* subjects were subjected to random noise transmitted to their temporal bone; while accelerations were measured in their left and right temporal bones (Hakansson et al., 1994). Between 14 and 19 frequencies were found for each subject, with considerable variability in the measurements. For example, the first resonance frequency ranged from 828 Hz to 1,164 Hz depending on the individual. Further *in-vivo* vibration studies are thus required to develop a more robust head substitute that takes into account additional soft tissues.

## 5 Conclusion

In conclusion, numerical and experimental analyses were carried out to establish the vibration modes of a newly designed cranial substitute. When considering only six measurement lines on the 3D-printed prototype of the substitute, more than 65% of experimental results are in agreement with the Finite Element Model of said substitute. The developed prototype and the FEM respectively have 8/13 and 10/13 natural frequencies in common with dry human skulls in the frequency range [0–10] kHz, with acceptable levels of errors. Hence, this new substitute could be used in the future as a first approximation of a human skull for vibration studies. After being filled with a brain matter simulant to be determined, it could be once again subjected to modal analyses before being subjected to high dynamic solicitations, such as blast loading, in order to investigate the cranial deflection injury mechanism.

## Data Availability

The original contributions presented in the study are included in the article/Supplementary Material, further inquiries can be directed to the corresponding author.
