# Supplementary material for: Design of a simplified cranial substitute with a modal behavior close to that of a human skull
Source: Front Bioeng Biotechnol. 2024 Mar 22;12:1297730. doi: 10.3389/fbioe.2024.1297730 (PMC10995299; doi:10.3389/fbioe.2024.1297730)
Supplement: Supplementary file 1 [file DataSheet1.PDF]

Supplementary Material

Design of a simplified cranial substitute with a close modal behavior as to a human skull

Natacha Elster*, Johanna Boutillier, Nicolas Bourdet, Pascal Magnan, Pierre Naz, Rémy Willinger, Caroline Deck

*** Correspondence:** Natacha Elster: Natacha.elster@isl.eu

1 Supplementary data A

The table presents a 2D comparison between the experimental mode shapes and the Finite Element Model of the skull substitute. The mode shapes are detected during rotations of the horizontal measurement lines along the y-axis and rotations of the vertical measurement lines along the x-axis. Color coding is the normalized magnitude of vibrations for each mode shape and each set.

Mode (n, m)	Experimental x-rotation	Computational	Experimental y-rotation	Computational
**1 (n=0, m=1)**	2102 ± 23 Hz	2008 Hz		
	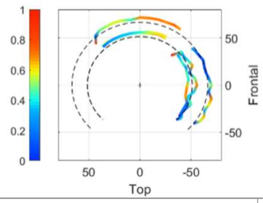	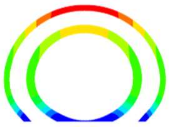		
**2 (n=1, m=1)**	2953 ± 26 Hz	2976 Hz	2953 ± 26 Hz	2976 Hz
	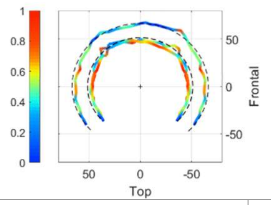	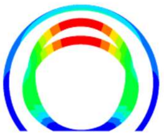	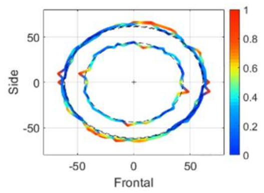	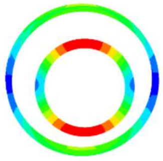
**3 (n=2, m=1)**	3139 ± 30 Hz	3004 Hz	3139 ± 30 Hz	3004 Hz
	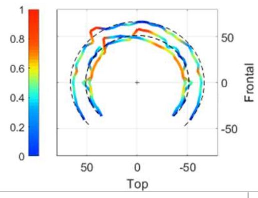	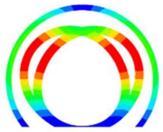	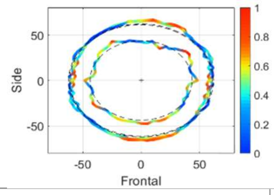	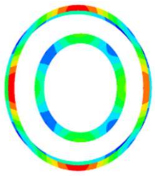
**4 (n=3, m=1)**	3512 ± 52 Hz	3475 Hz	3512 ± 52 Hz	3475 Hz
	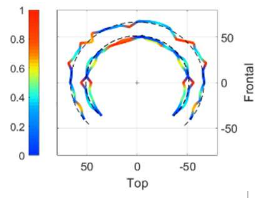	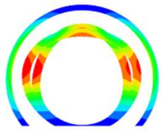	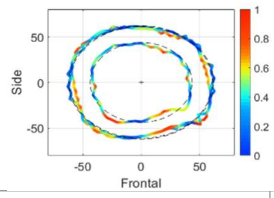	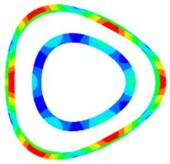
**5 (n=2, m=2)**	3610 ± 36 Hz	3636 Hz		
	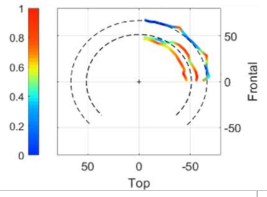	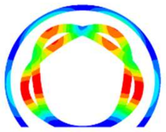		
**6 (n=1, m=3)**	3824 ± 83 Hz	3738 Hz		
	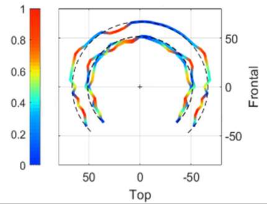	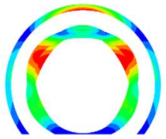		

Mode (n, m)	Experimental x-rotation	Computational	Experimental y-rotation	Computational
**7 (n=4, m=1)**			3954 ± 66 Hz	3844 Hz
			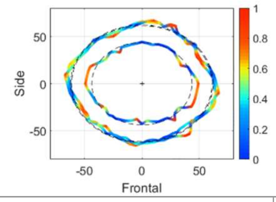	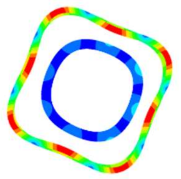
**8 (bi-axial)**	4145 ± 97 Hz	4278 Hz		
	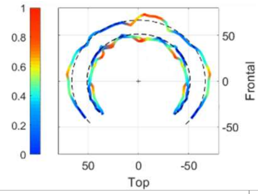	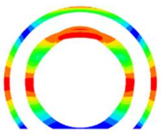		
**9 (n=5, m=1)**			4344 ± 79 Hz	4322 Hz
			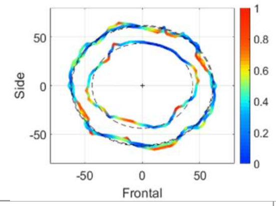	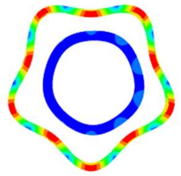
**10 (n=2, m=3)**	4371 ± 21 Hz	4308 Hz		
	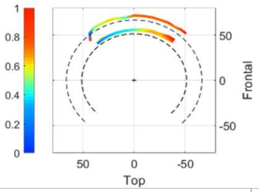	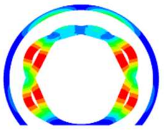		
**11 (n=4, m=2)**	4587 ± 78 Hz	4561 Hz	4587 ± 78 Hz	4561 Hz
	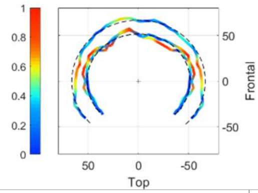	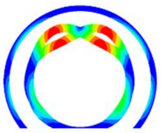	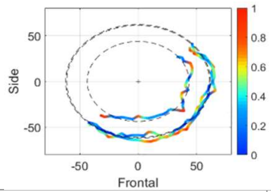	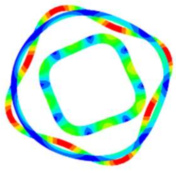
**12 (n=6, m=1)**			5168 ± 60 Hz	4985 Hz
			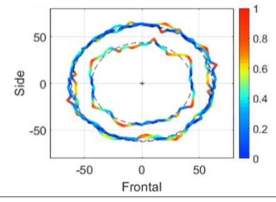	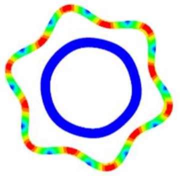

Mode (n, m)	Experimental x-rotation	Computational	Experimental y-rotation	Computational
**13 (n=3, m=3)**	5185 ± 98 Hz	4905 Hz		
	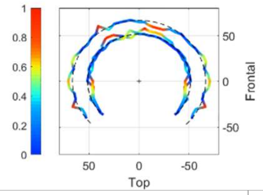	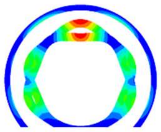		
**14 (n=2, m=4)**	5302 ± 27 Hz	5284 Hz		
	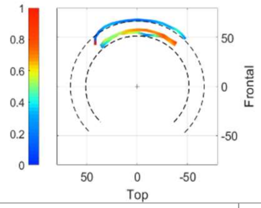	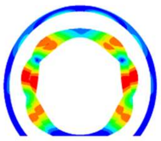		
**15 (bi-axial)**	5698 ± 116 Hz	5853 Hz		
	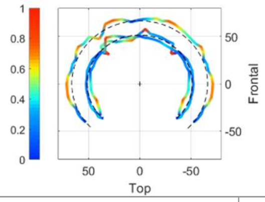	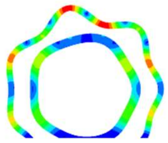		
**16 (n=6, m=2)**			6081 ± 95 Hz	6054 Hz
			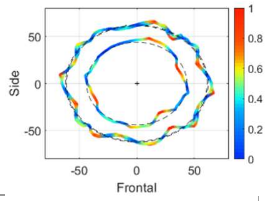	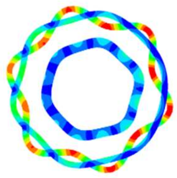
**17 (n=3, m=4)**	6126 ± 100 Hz	6098 Hz		
	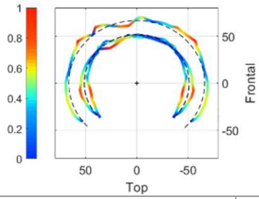	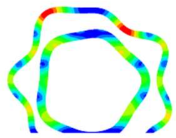		
**18 (n=5, m=3)**	6642 ± 156 Hz	6442 Hz	6642 ± 156 Hz	6442 Hz
	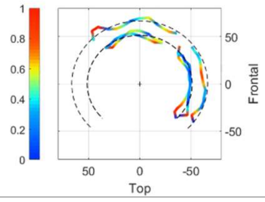	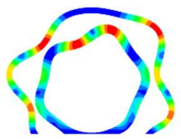	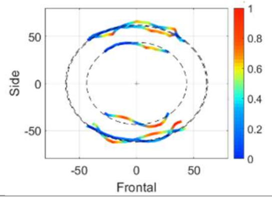	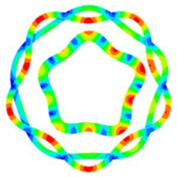

Mode (n, m)	Experimental x-rotation	Computational	Experimental y-rotation	Computational
**19 (n=4, m=4)**	6939 ± 13 Hz	6980 Hz		
	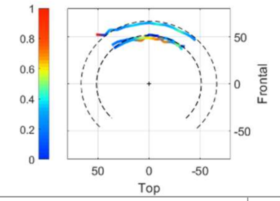	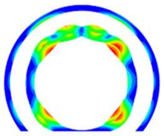		
**20 (n=1, m=5)**	7175 ± 113 Hz	7073 Hz		
	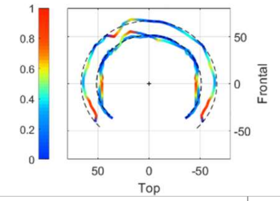	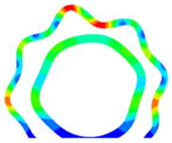		
**21 (n=6, m=3)**	7221 ± 60 Hz	7445 Hz	7221 ± 60 Hz	7445 Hz
	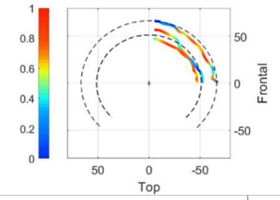	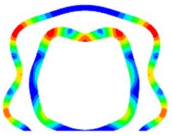	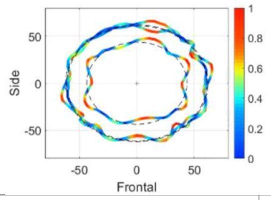	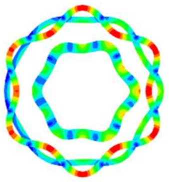
**22 (n=5, m=4)**	7914 ± 93 Hz	7988 Hz	7914 ± 93 Hz	7988 Hz
	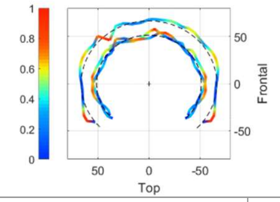	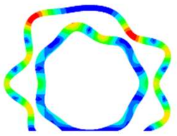	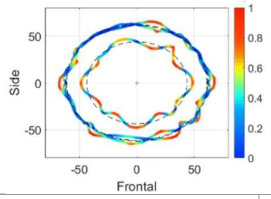	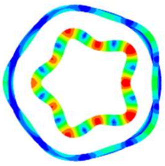
**23 (n=7, m=3)**			8448 ± 139 Hz	8598 Hz
			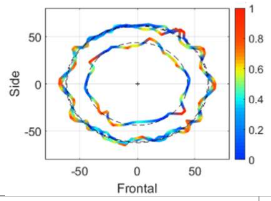	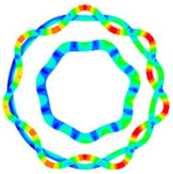
**24 (n=4, m=5)**	8604 ± 107 Hz	8673 Hz		
	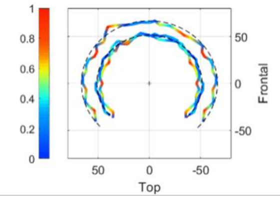	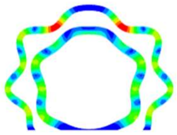		

<table>
<tr><th>Mode (n, m)</th><th>Experimental x-rotation</th><th>Computational</th><th>Experimental y-rotation</th><th>Computational</th></tr>
<tr><td>25 (biaxial)</td><td>8885 ± 4 Hz</td><td>9069 Hz</td><td></td><td></td></tr>
<tr><td></td><td>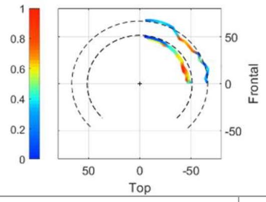
</td><td>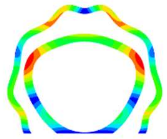</td><td></td><td></td></tr>
<tr><td>26 (n=6, m=4)</td><td></td><td></td><td>8921 ± 162 Hz</td><td>9124 Hz</td></tr>
<tr><td></td><td></td><td></td><td>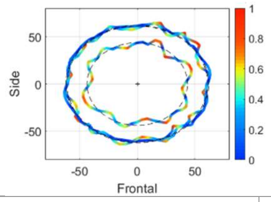
</td><td>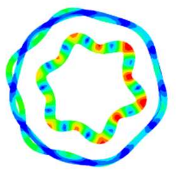</td></tr>
<tr><td>27 (n=1, m=8)</td><td></td><td></td><td>9223 ± 28 Hz</td><td>9348 Hz</td></tr>
<tr><td></td><td></td><td></td><td>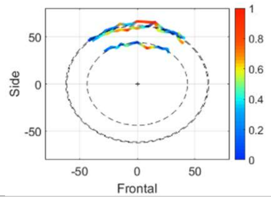
</td><td>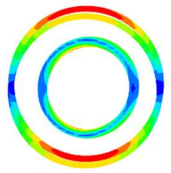</td></tr>
</table>
